# Amalgam as a Material for Filling Teeth

**Published:** 1883-11

**Authors:** G. W. Smith

**Affiliations:** Cincinnati, O.


### Amalgam as a Material for Filling Teeth.

BY DR. G. W. SMITH, CINCINNATI, O.

Read before the Mississippi Valley Dental Society, March, 1880.

In treating the subject of amalgam it is my purpose to consider first, the constituents of the fluid in the mouth, which has much to do with the disturbance that follows the use of these alloys as a filling material.

We know that food contains many organic substances, both of the hydro-carbonate and nitrogenous class, which, in the presence of warmth and moisture, will take on fermentation or putrefaction, as the case may be.

These processes, though closely allied, have each a distinct mode of action.

Fermentation has for its agent hydro-carbon, and is generally dependent on the presence of a putrescing nitrogenous body.

Its object is the re-arrangement of the atoms, forming generally not more than two compounds.

Putrefaction takes place only in bodies containing nitrogen, the atoms of which are liberated to re-unite according to their affinities, forming various compounds.

The oral cavity presents all conditions necessary to the first process; warmth and moisture, and not only the ptyalin of the saliva, which itself is a ferment, but very often decomposing nitrogenous matter is present.

There is, then, going on in the mouth from the presence of these nitrogenous substances, the formation of ammonia, from which nitrous acid may be formed in the mouth.

The nascent oxygen which, under such conditions, is always present will take its equivalent of hydrogen, from the ammonia, to form water; and thus one atom of nitrogen and hydrogen is set free.

The nascent oxygen will now combine with the nitr gen and the hydrogen, and in this way form nitrous acid.

We have, in addition to this, sulphurous and hydrochloric acids.

The sulphurous acid is formed from the sulphur, which is one of the constituents of the food.

Hydrochloric acid is formed in the mouth by common salt or chloride of sodium.

Acetic, lactic, and other acids are formed by the presence of glucose and cane sugars.

We have, entering into the formation of dental alloys, mercury, tin, silver, copper, gold, platinum, and other metals.

Dr. Ambler Tees gives us a formula, "which is highly recommended as affording excellent results."

The formula consists of:

| | |
|---|---|
| Tin | 40 dwt. |
| Silver | 24 " |
| Gold | 1 " |
| Platinum | 1 " |

You will readily perceive that tin and silver, with mercury, are the only metals that we can rely upon for this plastic compound.

When we take into account the small percentage of gold and platinum that enter into the formation of amalgams, it certainly becomes a debatable question whether these compounds should assume the name of gold and platinum alloys.

As mercury is the leading constituent in causing a union between these metals, it would be well, perhaps, for us to look into its properties, and see what effect it may produce on the human system.

Mercury is distilled from the ore, quicksilver, the chief supplies of which are obtained from Spain and South America.

This peculiar metal is an exception to all others, not only in its penetrating quality when brought in contact with other metals, but also in that it always remains in a fluid, movable condition, except when exposed to the severe cold of the Arctic regions.

It is known in the arts and commerce as quicksilver, and is distilled from its ore, just as water is distilled.

This metal, according to Faraday, has a peculiar effect on the tissues of the human system, for persons employed in the mines, or working with the metal, are generally afflicted with paralysis.

There are, perhaps, no metals employed or prepared for the dental profession for the restoration of broken-down dentures that should be so thoroughly investigated as those metals which are prepared and sold to dentists under the name of amalgams.

And yet we must acknowledge that we are daily dispensing this compound with but little or no concern as to the results which may follow its use as a material for filling teeth.

We are fully aware that the number of dentists advocating the use of these alloys are in the majority, who, without stopping to investigate the compound or the results that may follow its use, are simply content with the remuneration they receive for their skill and labor.

But let us, for a few minutes, dismiss our prejudices if we have any and see what it is, and how it may affect the nervous system, by chemical decomposition or metallic electricity. I deem it our privilege, as well as our duty as professional men, to study this amalgam question, and, if found to be a disturbing element as a filling material in the teeth, its use should, without further hesitation, be discontinued by the profession.

I believe that many dentists using amalgam in their practice at the present day would abandon its use if it could be made clear to their apprehension that this combination becomes a disturbing element when placed in the teeth.

It would be unprofessional for any one treating this subject to come before an intelligent society, like this, with a paper denouncing the profession, and those who use these alloys, as incompetent practitioners.

Not having any desire, then, to make an attack on those who use amalgam, we will simply call attention to the material, to ascertion whether or not we may have electrical currents arising from the presence of amalgam in the teeth.

Amalgam is formed by uniting tin, silver, copper and a small percentage of the nobler metals known as gold and platinum with mercury, and in these metals are found some of the active agents of the galvanic current.

It has been shown that the fluids of the mouth contain sulphurous, nitrous, and other acids, caused by the decomposition of hydro-carbons and nitrogenous substances present there, the solution of which excites molecular disturbance in the cell of the battery, composed of one part sulphuric acid to eight of water.

A chemical law is certainly here shown which pervades all atoms and molecular bodies—the great law of affinity.

Take, for example, two metals, silver and tin, and arrange them in the mouth that the tongue may keep them separated. As long as the metals are thus divided there will be no chemical action, but when the motion of the tongue brings them in close contact there is immediate action, and there will be given off from these metals chemical electricity.

A proof of this is in the metallic taste and the sharp, tingling sensation felt whenever the tongue comes in contact with the metals.

These conditions, it may be fairly concluded, are the primary principles of the voltaic battery, the electric current passing through the fluids of the mouth, in which the metals are immersed from the tin to the silver and again returning to the tin, forming a complete current of chemical electricity, by the dissolution of the metals acted upon by the acids contained in the saliva in which the metals are immersed.

Another simple experiment is made by placing a sheet of zinc in a weak solution of sulphuric acid, after which it should be rubbed over with mercury till it assumes a bright color; this may then be placed in a glass vessel, and opposite to it at some distance a piece of copper.

The glass vessel is then filled with a solution of sulphuric acid. There will be no molecular disturbance as long as the metals remain apart, but when connected by a conducting wire there is immediate action.

If we separate the wire at any point, leaving the ends in contact with the metallic bodies, the tongue will feel the same sensation, when brought in contact with the two ends of the wire, as that to which we have first alluded; leaving no room for doubt as to the fact that none of the metals which we have described can be immersed in this or even a weaker solution without manifesting a molecular disturbance.

If this phenomenon takes place whenever these metals are brought under these conditions, and these metals are the constituents of dental alloys, will we not have the same action when amalgam is exposed to the fluids of the oral cavity, as when these metals are immersed in the solution of the galvanic battery?

The principle is the same, and there is a law that cannot be suspended—" Like causes produce like results."

There is a prevailing idea with many, in reference to chemical action or metallic electricity, that there is an existing law of suspension, or negation, which renders the active properties of the saliva inert; a sort of antagonism that when dissimilar metals are placed in the mouth they are not acted upon by the acids contained in the saliva.

But as far as we know, science does not recognize any theory of this kind.

There was, however, quite a contest for a long time between those who maintained the theory of contact, and others who advocated the idea of chemical action as the cause of the development of voltaic electricity.

The former imagined that the mere contact of two dissimilar metals was the sole exciting cause. Faraday, however, by a series of masterly research, proved that such contact taking place, chemical action instantly ensued; and further—that electricity was evolved by chemical action in circumstances where contact was impossible.

These, with other facts, may be sufficient to prove that it would be impossible to retain the purest of these alloys in the teeth without galvanic action.

It being a fact then that no metals united by mercury can be present in the mouth without causing chemical electricity, it will be readily seen why these alloys should not be used as a filling material.

First, because the saliva contains more or less acid in its constituents, and is the same, in substance, as that found in the solution of the voltaic battery.

Secondly, because these alloys are composed of silver, tin, copper, and other metals, united by mercury, and never can exist in the fluids of the oral cavity without causing chemical decomposition or electric currents.

And what is the result?

These currents constantly vibrating along the tri-facial, or fifth pair of nerves, directly affect them, and by reflex action, the whole cranial system is disturbed, causing neuralgia, weak eyes, dizziness and affections of the auditory nerve.

But the question arises, how may we know that this continuous drumming or roaring sensation in the ears, which is the precusor of deafness, comes from the action of the electric current?

It has been proven, by actual test, that when the auditory nerve is freely stimulated by a galvanic current it will cause a roaring sensation through the head and ears.

And more, we are taught by electricians that strong currents are never proper about the face, neck or head, but should be applied cautiously, under all circumstances.

If science discovers to us these plain facts, and cases present themselves almost daily showing all the symptoms which have been mentioned, it is due these investigators that we, at least, put their researches to a practical test; and, if found true, give them the honor and attention due their labor.

Dentists have studied the art of relieving the suffering, and of repairing, if possible, the loss that dentures have sustained through the ravages of decay, but should one part of the system be pulled down to repair another part?

If it can be shown that amalgam strikes at the nerve centers (whatever good qualities it may otherwise possess) its presence in the mouth is certainly dangerous, and its use should be discontinued by the profession as a filling for decayed teeth.

As for my own limited knowledge regarding metals and their relations, there cannot be more than one metal present in the mouth at the same time without causing chemical action, let it be silver, tin, gold, or platinum.

We may, however, have oxydation but no electric manifestation, except two or more metals be present.

We have heard the reiteration from the vendors of these alloys, that "tons" of amalgam are used yearly for filling teeth, and no complaints are made about it.

I wish to express my denial of these groundless reiterations, coming as they do from the manufacturers of these alloys, who think only of the remuneration they receive from the sale of the material.

We have only to look at the increasing numbers who are daily consulting the occulists, the aurists, or the physicians for treatment to ascertain whether there is no complaint from amalgam fillings in the teeth.

"To-day many hundreds of agonizing neuralgias, weak eyes and ringing in the ears, would be radically cured by an application to the scientific dentist, rather than to a physician, who almost invariably resorts to opium in one form or other to lull the pain instead of removing the cause."

We are told by eminent writers upon the subject of galvanic stimuli, that in some cases of great torpor of the brain and of the cervical sympathetic ganglia, currents of considerable strength are necessary to cause vertigo, flashes of light and a metallic taste.

In other cases a feeble current from a single cup, for example, will cause very decided, even alarming effects.

This extreme degree of susceptibility is associated with a very sensitive and impressionable nervous system.

This extreme degree of susceptibility to galvanic action may also be acquired. Recent acute disorders, congestion and inflammation of the intra-cranial organs, have the effect of exalting the sensibility.

"That the existence of this extreme susceptibility is a contra-indication to the use of galvanism about the head and face is highly probable."

In conclusion, to express it all in a word, the appreciation of sensations of every conceivable character, varies greatly in different individuals.

There are persons in our city to-day, who have told me that they dare not put the point of a pen-knife to some of their teeth containing amalgam, without experiencing a quick, piercing sensation in the direction of the eye and ear; and the same steel point can be laid on a gold filling in the same mouth without any sensation or shock whatever.

In view of all these facts, relative to these alloys, can we fairly represent our worthy profession by dispening to humanity a compound that will not stand scientific investigation?

I fear not.

We hope the day is not far distant when the professors and teachers in our colleges will discourage the use of these alloys by their students, and urge them to a higher plane of professional skill and usefullness, which will not only be a credit to them in their high positions and the profession they represent, but a blessing to the race of mankind.

